# Early and Free Operation for Breast Cancer

**Published:** 1899-03-25

**Authors:** 


### EARLY AND FREE OPERATION FOR BREAST CANCER.

From a study of his own results, along with those obtained by Halsted, Butlin, and a variety of other operators, Watson Cheyne[1] draws certain important conclusions in regard to operations for breast cancer. Taking cases of all kinds together, not merely selecting favourable ones for operation, but operating on all cases in which operation is possible, it seems that we may reckon that about 50 per cent. of them will remain free from recurrence if the operations are carried out on the lines laid down by Heidenhain and Stiles; that even in the cases which do recur recurrence is considerably delayed, the patient enjoying a considerable period of good health; while the repeated operations which take place after the old plan of operating are now very rare, the recurrences when they do appear being usually in inoperable situations. The point of greatest importance, however, seems to be that the patient's chance depends much on what is done in the *first* operation. If this fails, either from imperfect removal of the disease, or on account of the extent of the disease in the first instance, further operation, however extensive, is seldom successful. Where a cancer is quite small patients and practitioners are very apt to be content in the first instance with a small operation, thinking that if recurrence takes place an extensive removal can be performed afterwards. This is an utter fallacy, and the performance of a partial operation in the first instance takes away, in Watson Cheyne's opinion, the patient's best and often her only real chance of getting rid of the disease.

[1] Lancet, Mar. 18.

